# Atherogenic indices in stroke patients: A retrospective study

**Published:** 2017-04-04

**Authors:** Rajaragupathy Sujatha, Subramanian Kavitha

**Affiliations:** Department of Biochemistry, PSG Institute of Medical Sciences and Research, Peelamedu, India

**Keywords:** Stroke, Lipid Profile, Atherogenic Indices

## Abstract

**Background:** Stroke makes a significant cause of morbidity and mortality worldwide. Although derangements in the lipid profile have been suggested as a risk factor for the development of stroke, various studies show inconsistent results on the association between lipid profile and stroke. A very few studies have commented on the status of lipid indices in stroke patients.

**Methods:** After obtaining ethical medical records of the study populations were analyzed, and data collected from patients admitted to the hospital with clinically diagnosed stroke and control group consisted of apparently healthy volunteers selected from the master health checkup department. Baseline characteristics and lipid profile parameters and the number of days of hospital stay for stroke patients were collected. Lipid indices were calculated using following formulae. Atherogenic index of plasma (AIP) = log triglyceride/high-density lipoprotein cholesterol (HDLc), Castelli’s Risk Index (CRI-I) = Total cholesterol/HDLc, CRI-II = Low density lipoprotein cholesterol/HDLc, atherogenic coefficient (AC) = (Total cholesterol−HDLc)/HDLc, and non-HDLc (NHC) = Total cholesterol–HDL.

**Results:** The study included 620 participants of which 290 were stroke patients and 330 healthy volunteers. 61% of stroke patients were hypertensives and 38% were diabetics 28% were both diabetic and hypertensives. In this study, the lipid parameters and the indices were significantly higher in stroke patients than the control group. Three indices, namely, CRI-I, AC, and NHC were found to be contributing to the risk of stroke significantly. There was no statistically significant correlation between the duration of hospital stay and lipid indices or individual parameters of lipid profile.

**Conclusion:** In this study, the atherogenic lipid indices were significantly higher in stroke patients compared to controls.

## Introduction

Stroke is defined as “an acute neurologic dysfunction of vascular origin with sudden (within seconds) or at least rapid (within hours) occurrence of symptoms and signs corresponding to the involvement of focal areas in the brain.”^1^ It is one of the leading causes of mortality and disability globally.

The prevalence of stroke in India varies in different regions of country, and the estimated prevalence rates increase from 0.3/1000 in < 45 years age group to 12-20/1000 in the 75-84 years age group.^2^

There are several studies in India determining risk factors of stroke. Goldstein, et al.^3^ described the various non-modifiable and modifiable risk factors of stroke. The non-modifiable risk factors include age, gender, ethnicity, and previous family history of stroke. The modifiable risk factors include hypertension, smoking, diabetes, asymptomatic carotid stenosis, atrial fibrillation, pre-existing cardiac disease, and hyperlipidemia.

Abnormalities of serum lipids have traditionally been regarded as a risk factor for coronary artery disease but not for stroke. However, the relationship between lipids and stroke was elucidated and it has been made clear that the risk of stroke and amount of carotid atheroma can be reduced with cholesterol-lowering medications.^3^ Thus, lipid profile abnormalities are found to have profound influences in the development and outcome of stroke.

It has been emphasized that in an attempt to optimize the predictive capacity of the lipid profile, several lipoprotein ratios or “atherogenic indices” have been defined. The various atherogenic indices are atherogenic index of plasma (AIP), Castelli Risk Index I and II (CRI), atherogenic coefficient (AC), and non-high density lipoprotein cholesterol (HDLc) (NHC).

AIP is based on two important parameters, serum triglyceride, and serum HDLc. The concurrent use of triglycerides and HDLc in this ratio reflects the multiple interactions among the metabolism of different lipoproteins and can be useful for predicting plasma atherogenicity. 

Based on the study by Dobiasova,^4^ we suggest that AIP values of -0.3 to 0.1 are associated with low, 0.10-0.24 with medium and above 0.24 with high cardiovascular risk.

It is calculated according to the following formula:

AIP = Log (serum triglyceride/serum HDLc).^4^

CRI-I and II are two important indicators of vascular risk, the predictive value of which is greater than the isolated lipid parameters. They are calculated as per the given formulae:

CRI-I = Serum total cholesterol/Serum HDLc,

CR-I II = Serum low-density lipoprotein (LDL)-cholesterol/Serum HDLc,^5^

AC is calculated as:

(Serum total cholesterol−Serum HDLc)/HDLc.^6^

NHC represents the cholesterol content present in all the atherogenic lipoproteins. It is calculated as the difference between total cholesterol and high-density cholesterol (serum total cholesterol–serum HDLc).

Studies have shown that lipid indices calculated from parameters of lipid profile were found to have better predictive capacity in cardiovascular disease.^7^ However, there were no conclusive studies to show the association of atherogenic indices in stroke patients. This study was conducted to assess the various lipid indices in stroke patients and also compare the same with healthy controls. 

Stroke is associated with serious morbidity and disability which contributes to the burden caused by stroke. Longer the duration of hospital stay in stroke patients, more is the severity of stroke and vice versa. This study also aimed to correlate these lipid indices with the duration of hospital stay in stroke patients which can be considered as an index of the severity of stroke.

## Materials and Methods

Ethical clearance was obtained from the Institutional Human Ethics Committee. The study group consists of patients admitted to the hospital with clinically diagnosed stroke during the period January 2015-2016. The control group consisted of apparently healthy volunteers selected from the master health checkup department.

The medical records of the study participants were analyzed, and data collected include age, gender, lipid profile parameters (total cholesterol, HDL, LDL and triglyceride levels), and the number of days of hospital stay for stroke patients.

All parameters were estimated using dedicated kits and reagents in autoanalyzer. Lipid indices were calculated using following formulae:

AIP = Log (serum triglyceride/serum HDLc),

CRI-I = Serum total cholesterol/serum HDLc,

CRI-II = Serum LDL cholesterol/serum HDLc,

AC = (Serum total cholesterol−serum HDLc)/serum HDLc,

NHC = Serum total cholesterol-serum HDLc.

All statistical analysis was performed with SPSS version 19. Data were expressed as mean ± standard deviation (SD). For comparison of variables, statistical test was done using Mann-Whitney U-test for skewed distribution and chi-square test for categorical variables. Odds ratio and 95% confidence interval was calculated. Factors found to be significant in the univariate analysis were subjected to logistic regression analysis. P < 0.050 was considered as statistically significant.

**Table 1:** Characteristics of the study population

**Characteristics**	**Cases**	**Controls**	**P**
Number of participants	290	330	
Age (year) (mean ± SD)	60.48 ± 13.61	58.78 ± 10.24	0.080
Gender [n (%)]			
Males	200 (69.0)	214 (64.8)	0.305
Females	90 (31.0)	116 (35.2)	
Hypertension [n (%)]	178 (61.4)	-	-
Diabetes mellitus [n (%)]	111 (38.3)	-	

## Results

A total of 620 participants were included in the study, out of which 290 were cases and 330 were controls. Age and gender matched controls were included in the study. The most commonly associated comorbid conditions related to stroke was found to be hypertension and diabetes mellitus. The characteristics of the study population are described in table 1.

The results of lipid profile and lipid ratios in the study population are given in table 2.

Among the 290 stroke patients, 251 (86.5%) had associated comorbid conditions such as hypertension, diabetes mellitus, coronary artery disease, atrial fibrillation, thyroid dysfunction, hyperhomocysteinemia and anemia. 

Cutoff levels of the atherogenic indices to assess cardiovascular risk were taken from previous studies.^4^^,^^8^ AIP value of < 0.1 was considered as low risk and ≥ 0.1 was considered high risk.^4^ CRI-I values of < 4 and CRI-II values of < 3 were taken as low risk.^8^ AC value of < 2 and NHC value of < 130 were considered to be low risk. 

Using these cutoff values, study participants were categorized as low- and high-risk individuals.

96.6% of stroke cases had AIP levels > 0.1 and only 81.5% of controls had AIP above the cutoff levels. 70.0% of cases had CRI-I levels > 4 and only 15.5% of controls had higher CRI-I levels. 59.7% of cases had CRI-II levels > 3 and only 13.6% of controls had higher CRI-II levels. 92.4% of cases had AC levels > 2 and only 44.2% of controls had AC above the cutoff levels. 53.4% of cases had NHC levels > 130 mg/dl and 11.8% controls had higher NHC levels. 

Chi-square test was performed to assess the significance between the two groups and the results are tabulated. Odds ratio was also calculated and the results are included in table 3.

Further logistic regression analysis (Table 4) was performed and three indices, namely, CRI-I, AC, and NHC were found to be contributing to the risk of stroke significantly.

There was no significant correlation between the duration of hospital stay and any of the lipid profile parameters or lipid indices among the stroke patients.

## Discussion

Stroke makes a significant cause of morbidity and mortality worldwide. There are several etiologies and risk factors which are associated with the development of stroke. Although derangements in the lipid profile have been suggested as a risk factor for the development of stroke, various studies show inconsistent results on the association between lipid profile and stroke.^9^

**Table 2:** Lipid profile parameters and lipid indices in study participants

**Parameter**	**Cases (mean ± SD)**	**Controls (mean ± SD)**	**P**
Serum cholesterol (mg/dl)	170.80 ± 44.00	144.10 ± 28.92	< 0.001*
Serum triglycerides (mg/dl)	140.50 ± 82.00	95.98 ± 37.25	< 0.001*
Serum HDL (mg/dl)	36.11 ± 10.40	47.37 ± 10.87	< 0.001*
Serum LDL (mg/dl)	116.23 ± 38.68	90.63 ± 29.06	< 0.001*
AIP	0.56 ± 0.27	0.26 ± 0.22	< 0.001*
CRI-I	4.99 ± 1.52	3.21 ± 1.16	< 0.001*
CRI-II	3.39 ± 1.26	2.06 ± 1.06	< 0.001*
AC	3.99 ± 1.50	2.21 ± 1.16	< 0.001*
NHC	134.78 ± 41.25	96.74 ± 30.32	< 0.001*

AIP: Atherogenic index of plasma; CRI: Castelli Risk Index; AC: Atherogenic coefficient; NHC: Non-high density lipoprotein cholesterol; HDL: High-density lipoprotein; LDL: Low-density lipoprotein; SD: Standard deviation

* P < 0.050 was considered as statistically significant.

**Table 3:** Association of atherogenic indices and stroke

**Parameter**	**Cases (%)**	**Controls (%)**	**P**	**OR**	**CI**
AIP	96.6	81.5	< 0.001*	6.34	3.18-12.65
CRI-I	70.0	15.5	< 0.001*	12.76	8.64-18.85
CRI-II	59.7	13.6	< 0.001*	9.36	6.32-13.86
AC	92.4	44.2	< 0.001*	15.35	9.44-24.95
NHC	53.4	11.8	< 0.001*	8.56	5.70-12.85

AIP: Atherogenic index of plasma; CRI: Castelli Risk Index; AC: Atherogenic coefficient; NHC: Non-high density lipoprotein cholesterol; OR: Odds ratio; CI: Confidence interval

* P < 0.050 was considered as statistically significant.

**Table 4:** Adjusted odds ratio using logistic regression

**Parameter**	**Adjusted odds ratio**	**CI**
CRI-I	6.470	12.52-16.58
AC	5.191	2.87-9.37
NHC	1.912	1.11-3.28

CRI: Castelli Risk Index; AC: Atherogenic coefficient; NHC: Non-high density lipoprotein cholesterol; CI: Confidence interval

Calculations of various lipid ratios and indices may show the existence of association if any. These indices include AIP, CRI, AC, and NHC. 

In this study, stroke was found to be more prevalent among men (69%) compared to women, which is consistent with many previous studies in which males have 25-30% higher risk of developing stroke.^10^ 61% of stroke patients had hypertension and 38% were diabetics 28% were both diabetic and hypertensives. 

Based on ATP III guidelines, 91% of stroke patients (n = 265) had alterations in one or more lipid parameters. All the parameters of lipid profile were significantly higher in cases when compared to controls. Our findings were inconsistent with studies by Shahar, et al.^11^ and Bowman, et al.^12^ which reported the lack of association between lipids and stroke. 

Lipid indices have been associated with increased cardiovascular risk in many previous studies.^4^^-^^6^ A very few studies have commented on the status of lipid indices in stroke patients.^13^^,^^14^ In the present study, the lipid indices were significantly higher in stroke patients than the control group. With inconsistent lipid profile parameters between stroke patients and control subjects, the lipid indices show a significant increase in stroke patients compared to controls. Even if lipid profile fails to show any risk, calculation of lipid indices will help to show the true risk existing between lipid abnormality and stroke development. 

Study participants were categorized using cutoff levels for the various atherogenic indices. It was found that stroke cases had statistically significant individuals in the high-risk group as shown in table 3.

Logistic regression analysis (Table 4) was performed and three indices, namely, CRI-I, AC, and NHC were found to be contributing to the risk of stroke significantly.

CRI-I was also a significant risk factor for stroke development which is similar to the study by Zhang, et al.^15^ which showed a positive association of CRI-I with the risks of total and ischemic stroke in both men and women. AC is a measure of cholesterol in LDL and very LDL fractions with respect to HDL. As AC value increases, the risk for developing cardiovascular diseases increase and vice versa. This study shows that higher levels of AC are a significant risk factor for stroke development. NHC was found to be a significant risk factor for the development of stroke (odds ratio-1.912). This finding was consistent with the study by Wu, et al.^16^ which stated that higher serum NHC is associated with increased risk for stroke independent of other potential confounding factors.

Further this study also assessed the severity of stroke patients by analyzing the duration of hospital stay. Pearson’s correlational analysis revealed that there was no statistically significant correlation between the duration of hospital stay and lipid indices or individual parameters of lipid profile. Patil and Raghuwanshi^17^ concluded that since all lipid parameters showed a considerable decrease on 7^th^ day, as the stroke severity decreased, it could be proportionally linked with the severity of stroke. However, there are no previous studies which show an association between the lipid indices and severity of stroke.

Hence, this study shows that lipid indices may help in assessing the risk of stroke if not the severity of the same. This study may also help guide future trials attempting to relate lipid alterations with the occurrence of stroke events.

## Conclusion

In the present study, the atherogenic lipid indices were significantly higher in stroke patients compared to controls. Three indices, namely, CRI-I, AC, and NHC were found to be contributing to the risk of stroke significantly. These can be easily estimated from routinely done parameters and is therefore a cheaper alternative to other costly diagnostic tests and modalities. The inclusion of these indices in routine clinical setup may help to identify at risk individuals and guide effective treatment modalities in stroke patients. 

***Limitations and Recommendation***

The study did not include the other comorbid conditions that are associated with stroke. Prospective studies can be undertaken to assess the various complications associated with stroke and its association with the various lipid indices.

## Notes:

**How to cite this article:** Sujatha R, Kavitha S. Atherogenic indices in stroke patients: A retrospective study. Iran J Neurol 2017; 16(2): 78-82.
